# Evaluating PI-RADS lesions and clinically significant prostate cancer in Black and Asian men: a PREVENT randomized clinical trial secondary analysis

**DOI:** 10.21203/rs.3.rs-6905600/v1

**Published:** 2025-07-04

**Authors:** Conor Driscoll, Nicole Handa, Mitchell Huang, Adam Murphy, Jim Hu, Edward Schaeffer

**Affiliations:** Northwestern University Feinberg School of Medicine; Northwestern University, Feinberg School of Medicine; Northwestern University Feinberg School of Medicine; Northwestern University Feinberg School of Medicine; Weill Cornell Medical College; Northwestern University

**Keywords:** Ethnic and racial minorities, clinical trial, disparities, MRI, prostate cancer

## Abstract

**Purpose:**

Non-White patients are poorly represented in prostate cancer trials. MRI PI-RADS scoring was developed in primarily White populations, but prostate cancer differs in non-White men. We aimed to explore differences in PI-RADS calibration for Asian and Black men.

**Materials and Methods:**

This is a secondary analysis of PREVENT, a multi-institutional study of infection rates for transrectal vs. transperineal biopsy. We compared cancer detection for self-identifying Asian and Black men. We compared detection rates on a per-person basis, stratified by index PI-RADS lesion, to White men, using Fisher’s exact and logistic regression.

**Results:**

Of 665/752 trial patients with PI-RADS 3–5 lesions, 88 (13%) were Black and 36 (6%) were Asian. Black men were younger at diagnosis with increased rates of overall (70% vs. 43%%, *P*=0.004) and clinically significant prostate cancer (60% vs. 27%, *P*=0.003) and Asian men had decreased rates of overall (0% vs. 47%, *P*=0.004) and clinically significant prostate cancer (0% vs. 27%, *P*=0.003) in PI-RADS 3 lesions compared to White men. On multivariable regression, Black men with PI-RADS 3/4 lesions had higher odds of overall (OR 1.17, *P*=0.009) and clinically significant prostate cancer (OR 1.20, *P*=0.004) and Asian men had lower odds of overall (OR 0.79, *P*=0.01) but not clinically significant prostate cancer (OR 0.94, *P*=0.5).

**Conclusions:**

Black men with PI-RADS 3/4 lesions had 20% higher odds of clinically significant prostate cancer than White men while all PI-RADS 3 lesions in Asian men were negative. These findings suggest PI-RADS may require differential interpretation when assessing prostate cancer risk in non-White men.

**Source of Funding:**

Supported by the NCI (5R01CA241758-05).

**Trial Registration:**

Registered at ClinicalTrials.gov (NCT04843566, https://clinicaltrials.gov/study/NCT04843566).

## Introduction

Prostate cancer (PCa) is the most common solid malignancy in men, affecting 1 in 8 men in their lifetime.^1^ There is a significant differential in PCa disease burden by race in the United States with Black men experiencing younger age at PCa diagnosis, higher grade PCa at diagnosis, and higher PCa-specific mortality.^2^ Multiple theories exist in the literature as to why this health disparity exists, including access to care, genetic variations (such as CDKN1B deletions), and Vitamin D related molecular mechanisms.^3–6^ While many of these may contribute to the differences in PCa outcomes for Black men, none of these have been able to fully explain the disparity.

Another reason that has been well-documented across multiple fields of medicine, is that randomized controlled trials (RCT) tend to have poor accrual of non-White patients, which can prevent findings from being validated across racial groups and possibly lead to unintentional mismanagement of non-White patients.^7,8^ Over the past several decades, there has been significant attention directed to refine the evaluation of PSA testing to better balance detection of clinically significant cancer with harms associated with over-diagnosis. Although algorithms differ from institution to institution, many algorithms use serum PSA level and PI-RADS lesions on MRI as the main factors that determine when to biopsy a patient. Given that PSAD and PI-RADS were validated in a predominantly White population^9^, it is possible that mis-calibration of these clinical factors could be contributing to PCa outcome disparities for Black men.

Similarly, Asian men remain under-represented in pivotal PCa trials.^10^ While the known disparities for Black men have generated a significant arm of research into the cause, there is a much smaller body of literature surrounding PCa in Asian men, a demographic that is growing rapidly in the United States. What data does exist, however, demonstrates lower rates of grade group (GG) 1 PCa detection and fewer positive biopsies for PI-RADS 3 lesions compared to non-Asian peers, suggesting a possible mis-calibration of current risk stratification tools for Asian men as well.^11^

The PREVENT trial was a multi-institutional randomized controlled trial which compared infectious outcomes between transperineal (TP) and transrectal (TR) prostate needle biopsy (PNB). The study captured self-identified race and MRI lesion data with self-identifying Asian men representing 4.9% and self-identifying Black men representing 13.4% of the overall trial cohort, respectively.^12^ Here, we analyze biopsy outcomes for both self-identifying Black and Asian patients in the PREVENT trial with the hypothesis that MRI may not be appropriately calibrated for these populations.

## Materials and Methods

### Study Participants

Patients randomly allocated to the PREVENT trial’s TP vs. TR PNB comparison were eligible for study. The first efficacy results from this comparison have been published previously.^12^ Briefly, from March 2021 through May 2023, patients were recruited at ten centers and were eligible for enrollment if they had not undergone prior PNB, had an elevated PSA level and/or abnormal digital rectal examination (DRE), and had suspicious prostate magnetic resonance imaging (MRI) characteristics (Prostate Imaging Reporting and Data System, version 2.1^13^ scores 3–5). They were then randomized 1:1 to TP PNB without antibiotic prophylaxis or TR PNB with targeted antibiotic prophylaxis. All patients underwent multiparametric MRI (mpMRI) per study protocol prior to randomization. Reviewers were blinded to treatment allocation and outcomes as previously reported.^12^ A small number of participants had biopsy without MRI (claustrophobia and metal prosthesis) and were included in the original trial but were excluded from this secondary analysis as the primary outcome of this analysis is predicated on presence or absence of mpMRI PI-RADS lesions. The exclusion criteria were acute prostatitis in the last 6 months or any current bacterial infection requiring antibiotic treatment. All patients provided written informed consent. The trial is registered at ClinicalTrials.gov (NCT04843566), funded by the NCI (5R01CA241758-05) and had full regulatory, national ethics committee, and local site approval. Full details of the PREVENT trial protocol is provided in Supplementary Methods 1. As part of the trial, race data was collected via self-reporting and used for this secondary analysis.

### Outcomes

The PREVENT trial comparison’s primary outcome measure was infection rate by PNB approach but here we focus on overall and clinically significant PCa (csPCa) rates by race. Rates of overall and csPCa defined as Gleason Grade Group ≥2 detection on a per-person basis were compared based on self-identified race, stratified by PI-RADS score of the person’s index lesion. As such, only those patients with PI-RADS ≥3 lesions on MRI were included.

### Statistical Analysis

For continuous variables, Shapiro-Wilk test was used to assess normality. We then used Kruskal-Wallis and one-way ANOVA tests as appropriate to compare continuous variables across racial groups; for comparisons that were found to be significantly different across racial groups, we performed subsequent pairwise Wilcoxon or t-tests, respectively, with Bonferroni correction applied to adjust for multiple comparisons. Fisher’s exact test was used to compare categorical variables.

For the subset of patients who had a PI-RADS 3 or 4 lesions (considered to be intermediate to high risk under PI-RADS), we created separate multivariable logistic regression models with the outcome of interest as either any PCa detection or csPCa. We used these models to assess for possible associations between patient reported race and these outcomes of interests. Variables were included in our model by consensus from our study team based on likelihood of association with cancer detection.

Cutoff for clinical significance for all analyses was p < 0.05. All statistical analysis was conducted in R version 4.4.2.

## Results

At the completion of enrollment, the PREVENT trial included 665 patients from March 2021 through May 2023 who had a PI-RADS lesion visible on MRI. The CONSORT diagram from the original trial is included as Supplementary Fig. 1. Of this cohort, 414 (62%) patients self-identified as White, 88 (13%) as Black, 36 (6%) as Asian, and 125 (19%) declined to report their race or identified as “Other.” Black men were significantly younger at PCa diagnosis (*P* = 0.001) with higher PSA (*P* = 0.02) and higher PSA density (PSAD) (*P* = 0.01). Baseline patient characteristics, including prostate cancer risk factors are summarized in **Table 1**.

**Figure 1:**
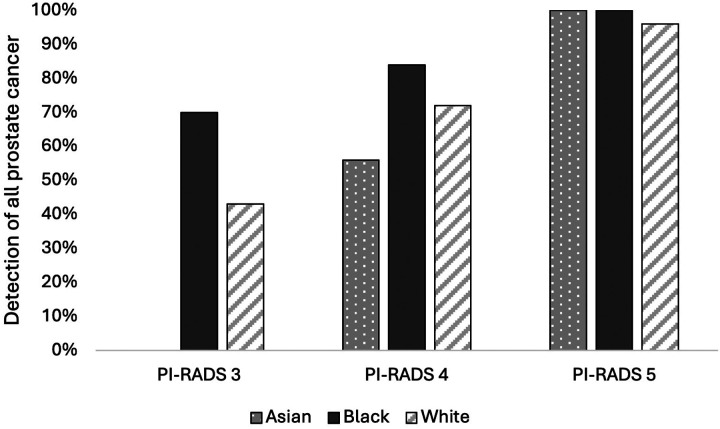
Detection rates of overall prostate cancer on biopsy stratified by PI-RADS and race.

**Figure 2:**
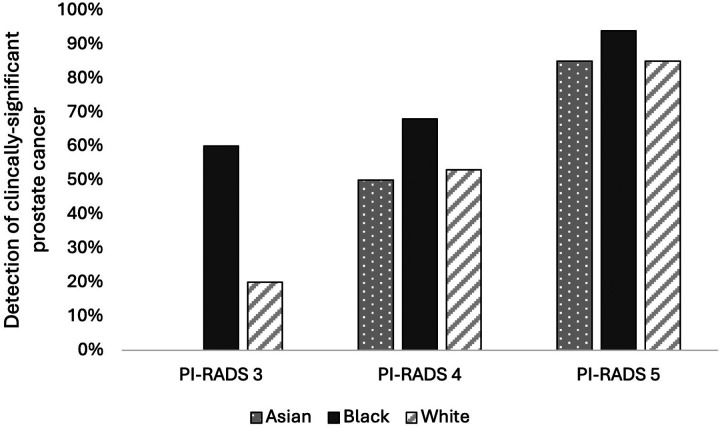
Detection rates of clinically significant prostate cancer on biopsy stratified by PI-RADS and race.

When assessing for cancer detection, there was a statistically significant increase in rates of overall PCa (70% vs. 43%%, *P* = 0.004) (Fig. 1) and csPCa (60% vs. 27%, *P* = 0.003) for Black patients with PI-RADS 3 lesions compared to White patients (Fig. 2) (**Table 2**). On logistic regression controlling for age, biopsy approach, and PSAD, Black men with PI-RADS 3 or 4 lesions had higher odds of overall PCa (OR 1.17, *P* = 0.009) and csPCa (OR 1.20, P = 0.004) detection than White men with PI-RADS 3 or 4 lesions (**Table 3**).

For Asian men, there was a statistically significant decrease in rates of overall PCa (0% vs. 47%, *P* = 0.004) (Fig. 1) and csPCA (0% vs. 27%, *P* = 0.003) (Fig. 2) (**Table 2**). On logistic regression controlling for age, biopsy approach, and PSAD, Asian men with PI-RADS 3 or 4 lesions had lower odds of overall PCa (OR 0.79, *P* = 0.01) but not csPCa (OR 0.94, *P* = 0.5) than White men with PI-RADS 3 or 4 lesions (**Table 3**).

## Discussion

In this secondary analysis of the first multicenter, randomized trial comparing novel, office-based transperineal prostate biopsy with the traditional transrectal approach, we found increased detection of overall PCa and clinically significant PCa in Black men with a PI-RADS 3 lesion compared to non-Black men. We also found decreased detection of overall PCa and clinically significant PCa in Asian men with a PI-RADS 3 lesion compared to non-Asian men.

These findings in Black men suggest that the PI-RADS system in its current form is not appropriately calibrated for PCa risk assessment in Black men. Our data shows that Black men with a PI-RADS 3 or 4 lesions are 20% more likely to harbor csPCa than their non-Black counterparts, suggesting that a PI-RADS 3 or 4 lesion should be treated as a more concerning finding in this patient population. Furthermore, when looking at the data globally, the csPCa detection rate for a PI-RADS 3 lesion in a Black man exceeded the csPCa rate for PI-RADS 4 lesion in White patients, which in our study was 70%. This suggests that PI-RADS 3 lesions in Black men should be treated like PI-RADS 4 lesions in White men from a risk stratification perspective. While this simple “upgrading” of intermediate risk PI-RADS lesions for Black men is a certainly a compelling and facile clinical heuristic, it should not be a substitute for a more systematic and nuanced recalibration of the PI-RADS system.

Given that most current biopsy algorithms weigh a PI-RADS 4 differently than a PI-RADS 3 lesions for determining pre-test probability, it may explain why there is often a delay in diagnosis for Black men which in turn creates a racial disparity in PCa outcomes. Previous studies have suggested that differences in dynamic contrast enhanced MRI parameters exist between Black and White men^14,15^, which may explain the different behavior of PI-RADS lesions found in our study. Zabihollahy et al. (2024) performed a quantitative mpMRI study of PCa in Black and White patients and similarly found that there was an increased risk of csPCa in PI-RADS 3 and 4 lesions in Black men, along with higher rates of csPCa in the peripheral zone and posterior aspect of the prostate.^16^ Multiple other studies agree with this finding of increased rate of csPCa despite equivalent PI-RADS lesion in Black men^17,18^, although there are some studies that found that the PI-RADS 2.0 system underperforms in Hispanic men relative to White men but no difference between Black and White men.^9^ Although not entirely novel, our findings provide the highest level of evidence of this relationship, as this is the first data from a randomized controlled trial compared to non-randomized data in prior studies.

Further, in this study Black patients had significantly higher PSAD at time of enrollment compared to non-Black patients. It has previously been well documented in the literature that Black men have higher PSAD compared to non-Black men. There is also evidence that using the Prostate Health Index (PHI) may function better in Black men, who typically present with larger prostates and higher PSAD.^19,20^ Other studies have shown that biparametric MRI (bpMRI) performs as well as mpMRI in Black men^21^, a subject which was not explored in this study but provides a promising framework for further study.

Given that this is a randomized trial, this also represents the highest quality data to date in the literature evaluating PI-RADS performance in Asian men. None of the Asian men with PI-RADS 3 lesions in this study were diagnosed with PCa. While these results suggest that the risk of PCa may be overestimated using the PI-RADS system, it is important that they are interpreted in the context of a small sample size with only 7 patients. It is hard to know if these findings would be validated in a larger sample size. However, it is consistent with previous literature that Asian men have lower risk of csPCa per equivalent PI-RADS lesion compared to non-Asian men.^11,22^ While the two Gross et al. studies performed their analysis on Asian American men specifically, other cohort studies in Asia have demonstrated similar findings with rates of csPCa detection around 10% in PI-RADS 3 lesions, which is well below our findings of 28% in this study for White patients.^23,24^

Historically, Asian men have a lower prostate cancer incidence relative to other men in Western countries.^25,26^ While there are multiple explanations hypothesized for this, ranging from the protective effect of a soy-based diet to differences in nationalized screening protocols, there is no agreed upon explanation for this consistent finding.^25,27^ One major component to this difference may be that Asian/Pacific Islander men only account for 1.5% of all patients in PCa clinical trials per a 2023 meta-analysis.^28^ Given this disparity in representation, it is unsurprising that the risk assessment calculators derived from European trials, such as the European Randomized Trial for Screening of Prostate Cancer (ERSPC), underperform in Korean men relative to the Seoul National University Prostate Cancer Risk Calculator or that a universal PSA screening program in Japan yielded a ten-fold lower rate of PCa diagnosis than similar universal screening programs in other countries.^29,30^. With 5% of our trial population self-identifying as Asian, our data contains a more representative population of Asian men relative to the US population and provides the highest representation in a PCa randomized trial to date.

This study does have a couple of limitations. First, including only patients from the original PREVENT trial with PI-RADS 3 or greater lesions on mpMRI, the sample size of Asian and Black men was relatively small. Despite this, the distribution of race in our cohort closely reflects national demographic data and the rate of non-White patients is well above the representation in most previous clinical trials. Second, this study was completed with mpMRI as the imaging modality, which is not feasible in every clinical setting secondary to availability and/or cost. We believe that our findings would remain true even with bpMRI as the imaging modality of choice and hope to validate these findings in a similar patient population with bpMRI. Third, the PREVENT trial was powered to detect differences in infection rate, not cancer detection. As such, it may be underpowered to detect differences in cancer detection. Yet, despite this limitation we were able to identify clinically meaningful differences in cancer detection by race.

## Conclusions

Black men with PI-RADS 3 or 4 lesions had 20% higher odds of csPCa detection than their White counterparts, even after adjusting for other risk factors. By contrast, all PI-RADS 3 lesions in Asian men were negative. These findings suggest that PI-RADS, which was validated in a population of men of predominantly European descent, may have poor calibration when assessing csPCa risk in Black and Asian men. This could be related to greater prostate cancer prevalence or more aggressive prostate cancer among Black men and contribute to unnecessary biopsies in Asian men. The PI-RADS classification system should be systematically re-evaluated considering these findings to ensure adequate validation amongst different racial groups.

## Supplementary Material

Supplementary Files

This is a list of supplementary files associated with this preprint. Click to download.

- Logisticregressionmodelformula.docx
- BRANYApprovedBiopsyRCTProtocolv5.82025.01Finalcopy.pdf
- PCPDPREVENTBlackAsianSupplementaryMaterial.docx
- PCPDPREVENTAsianBlackCONSORTChecklist.doc
- PCPDSAPPREVENTBlackAsian.docx
- Table1PCPDPREVENTAsianBlack.xlsx
- Table2PCPDPREVENTAsianBlack.xlsx
- Table3PCPDPREVENTAsianBlack.xlsx

## Data Availability

Study protocol and statistical analysis plan are fully available in the text and supplement. After de-identification, individual participant data that underlie the study results and analytic code will be available immediately upon publication with no end date to researchers who provide a methodologically sound proposal. Proposals should be directed to e-schaeffer@northwestern.edu. To gain access, data requestors must sign a data access agreement.
